# Supplementary material for: Single-cell atlas of the first intra-mammalian developmental stage of the human parasite Schistosoma mansoni
Source: Nat Commun. 2020 Dec 18;11:6411. doi: 10.1038/s41467-020-20092-5 (PMC7749135; doi:10.1038/s41467-020-20092-5)
Supplement: Supplementary file 1 — Supplementary Information [file 41467_2020_20092_MOESM1_ESM.pdf]

Supplementary Information for

Single-cell atlas of the first intra-mammalian developmental stage of the human parasite *Schistosoma mansoni*

Carmen Lidia Diaz Soria[1]#, Jayhun Lee[2,3]#, Tracy Chong[2,3], Avril Coghlan[1], Alan Tracey[1], Matthew D Young[1], Tallulah Andrews[1], Christopher Hall[1], Bee Ling Ng[1], Kate Rawlinson[1], Stephen R. Doyle[1], Steven Leonard[1], Zhigang Lu[1], Hayley M Bennett[1], Gabriel Rinaldi[1]*, Phillip A. Newmark[2,3,4]*, Matthew Berriman[1]*.

Affiliations

1 Wellcome Sanger Institute, Wellcome Genome Campus, Hinxton, Cambridgeshire, UK
2 Regenerative Biology, Morgridge Institute for Research, Madison, WI, USA
3 Howard Hughes Medical Institute, University of Wisconsin–Madison, WI, USA
4 Department of Integrative Biology, University of Wisconsin-Madison, Madison, WI, USA

#Equal contribution: Carmen L. Diaz Soria, Jayhun Lee
*Co-corresponding authors: Gabriel Rinaldi (gr10@sanger.ac.uk), Phillip A. Newmark (PNewmark@morgridge.org), and Matthew Berriman (mb4@sanger.ac.uk)

Table of Contents

Supplementary Figures 1-12

Supplementary References

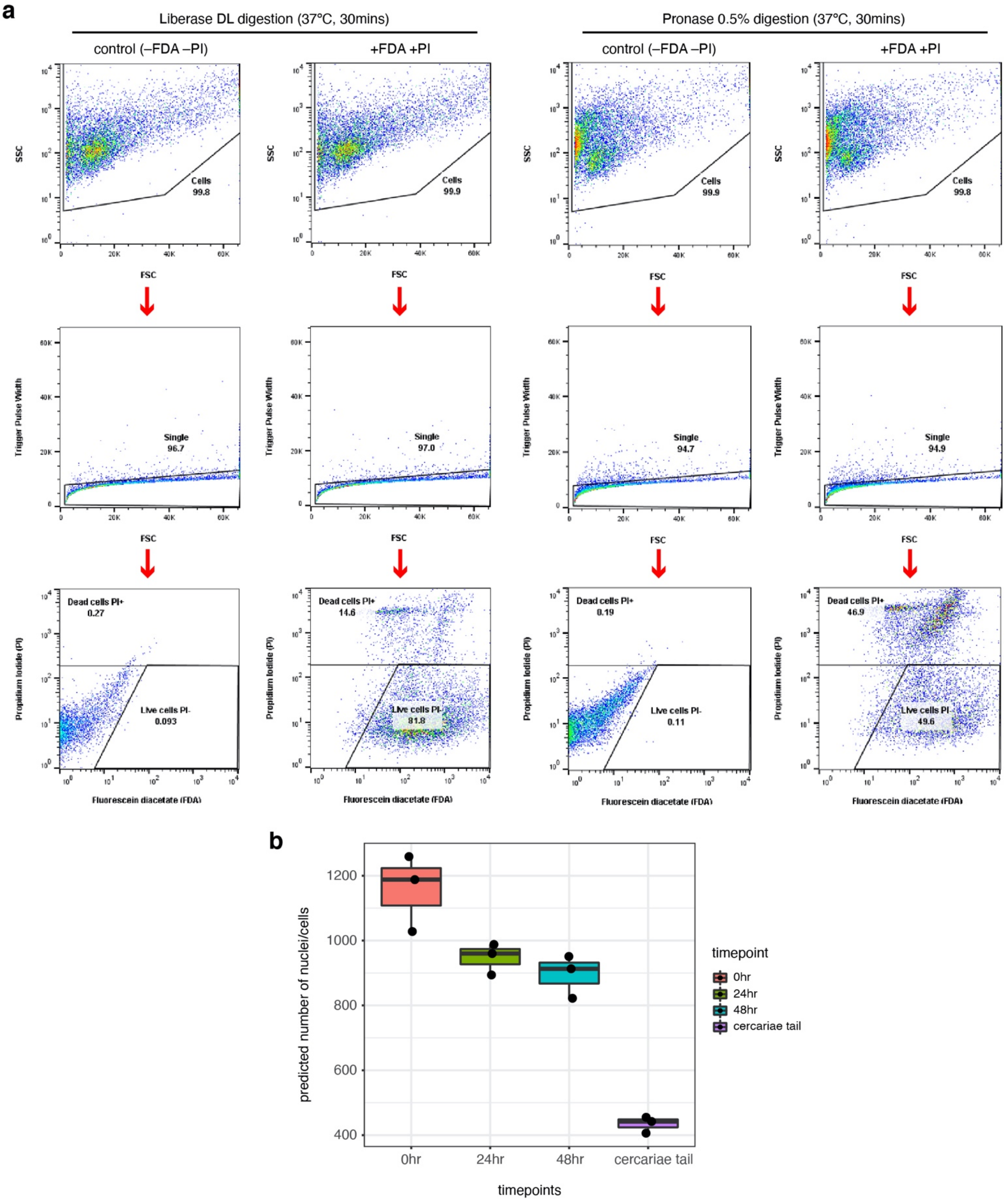

Supplementary Figure 1. (a) Comparison between protocols to dissociate schistosomula. Flow cytometry-based assessment of dissociation with either Liberase DL (750µg/ml) (left) or Pronase 0.5%

(right) revealed that the former led to more live cells than the latter. **(b)** Predicted number of cells that comprise a schistosomulum transformed *in vitro*. The bar chart shows the number of cells counted in schistosomula immediately after mechanical transformation (0 hr, i.e. cercaria head), after one day (24 hr) and two days (48 hrs) in culture. 'Tail' represents the number of cells counted in the tail detached from the cercaria during the mechanical transformation. Center lines show the medians; box limits indicate the 25th and 75th percentiles as determined by R software; whiskers extend 1.5 times the interquartile range from the 25th and 75th percentiles, outliers are represented by dots. n = 3 sample points.

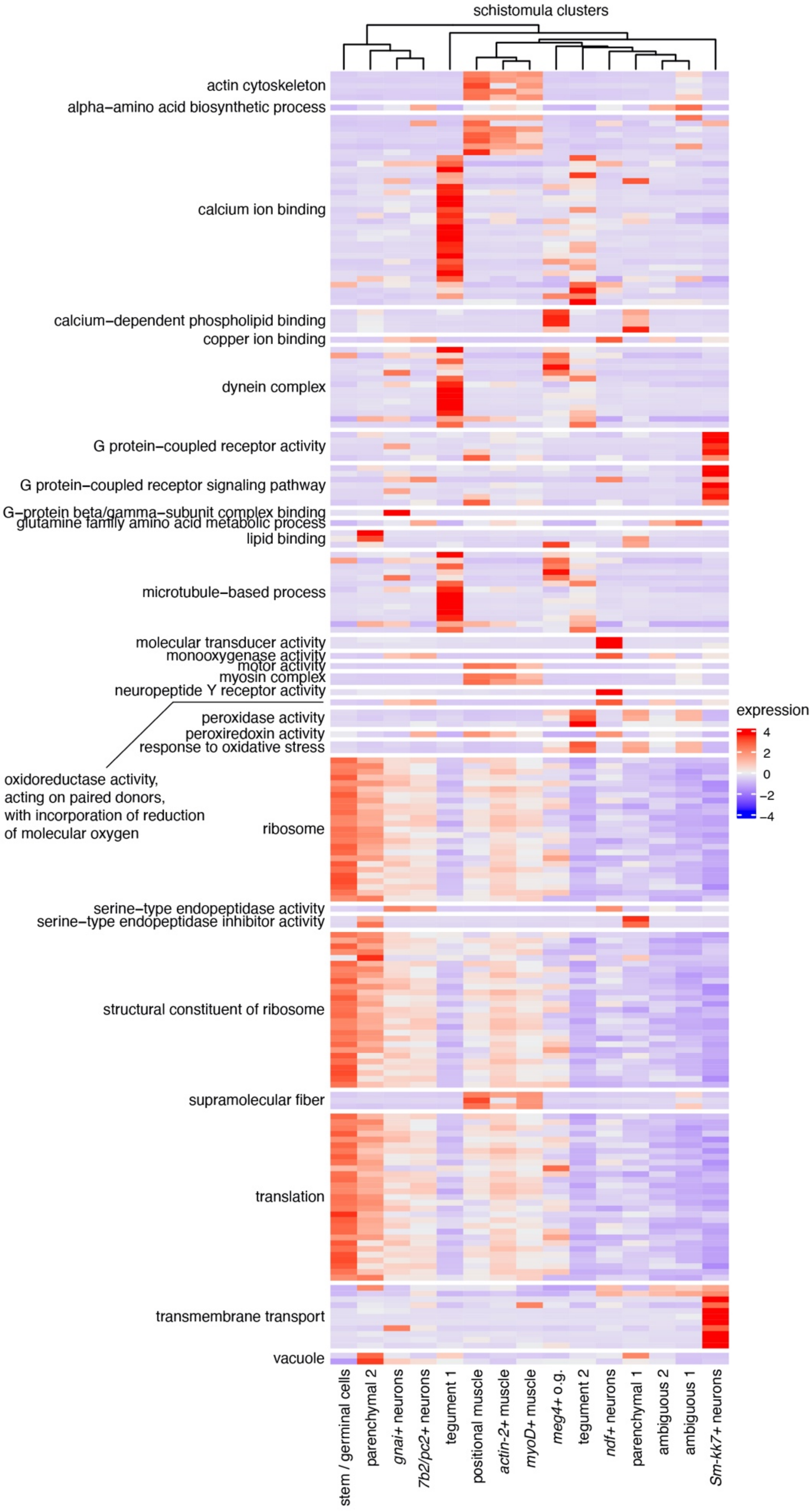

Supplementary Figure 2. Heatmap of differentially expressed genes between *S. mansoni* populations with representative GO Biological Process terms. Most populations show a coherent set GO terms that set them apart from other clusters. Ambiguous 1 and 2 are the exception, where no coherent set of GO terms are found.

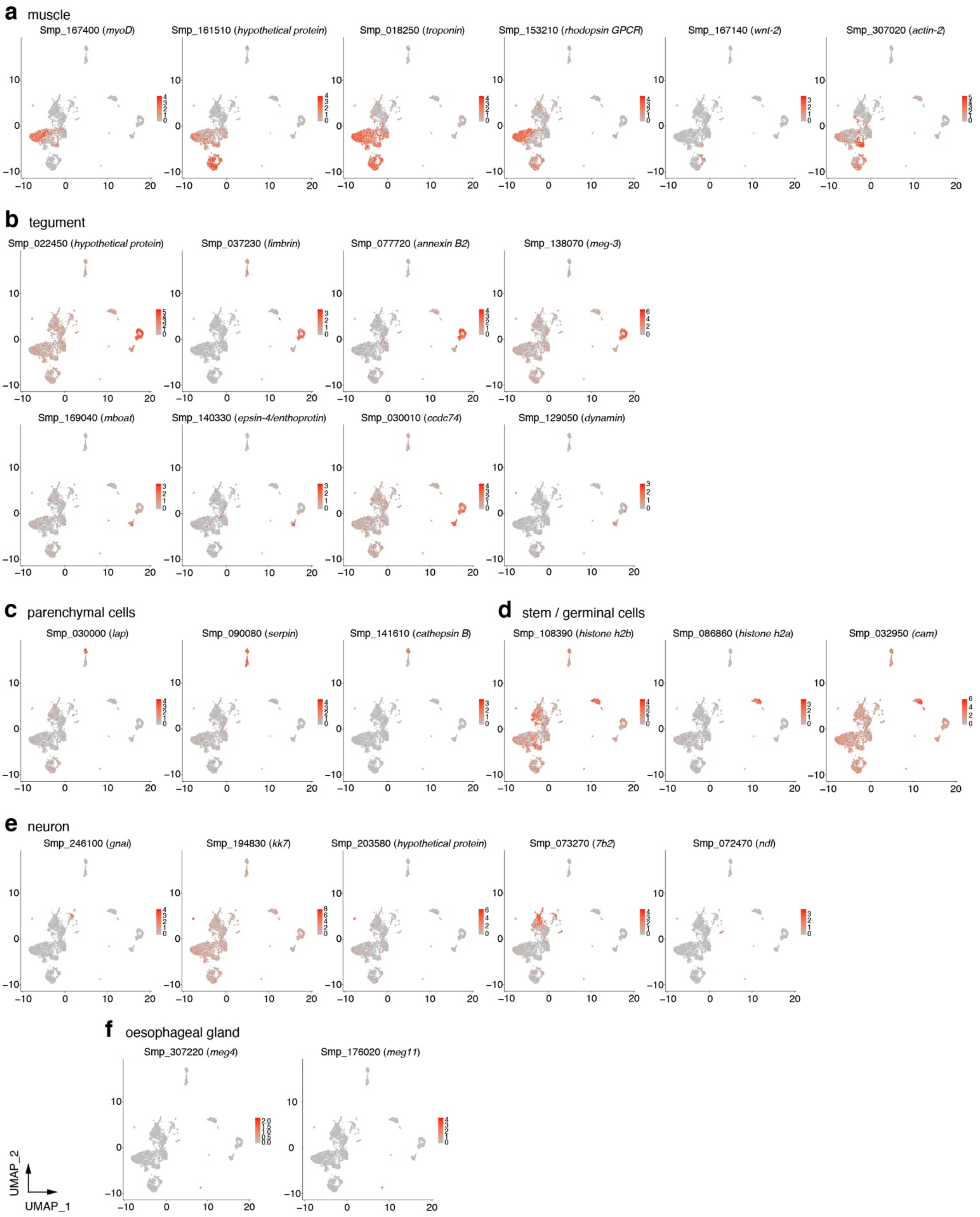

Supplementary Figure 3. UMAP plots showing the expression of representative marker genes for each

of the populations. All markers shown have been validated by FISH except for Smp_072470 (*ndf*) and Smp_176020 (*meg11*) **(a)** Muscle markers. **(b)** Tegument markers. **(c)** Parenchymal markers. **(d)** Stem markers. **(e)** Neuronal markers **(f)** Oesophageal gland markers.

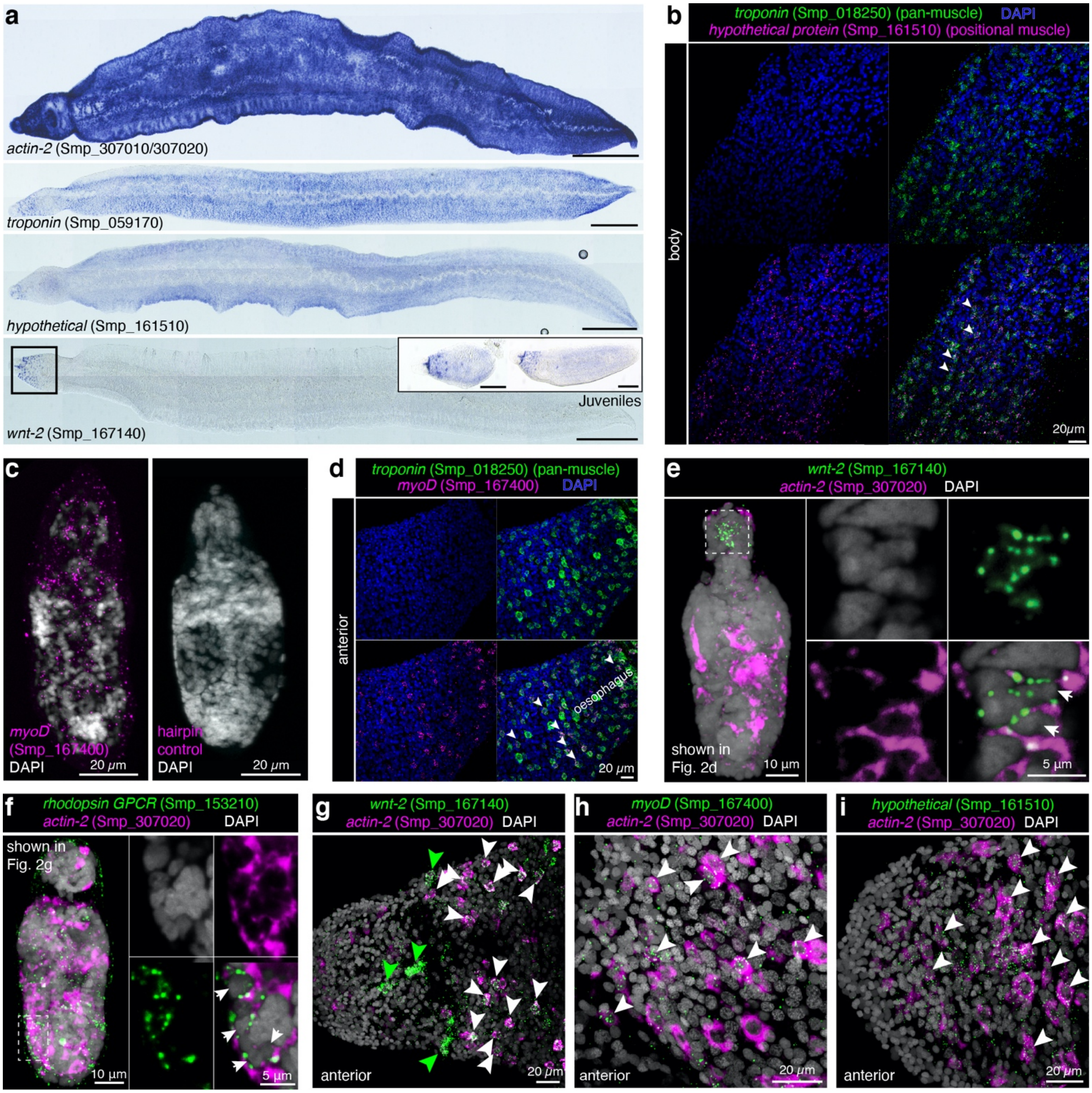

Supplementary Figure 4. (a) WISH using indicated markers and signalling molecules enriched in a subset of muscle cells in adult schistosomes. For *wnt-2*, the boxed region is shown in Fig. 2e. On the right, WISH experiment shows that *wnt-2* expression is conserved in the anterior end of juvenile parasites collected from mice 3 weeks post-infection. **(b)** Double FISH of Smp_161510 and a pan-muscle marker *troponin* (Smp_018250) in the mid-body region of the adult worm. **(c)** *In situ* hybridisation chain reaction (HCR) for *myoD* mRNA expression in a schistosomulum, MIP. Left Panel: *myoD* is expressed in cells that may form part of the body wall musculature. Right Panel: No probe control. Scale bar: 20um. **(d)** Double FISH of *myoD* (Smp_167400) and a pan-muscle marker *troponin* (Smp_018250) in the anterior region of adult worm. Majority of *myoD* expressing cells also express troponin (examples shown in white arrowheads). (**e-f**) Double FISH of *actin-2* (Smp_307020) and **(e)** *wnt-2* (Smp_167140) or **(f)** *rhodopsin*

GPCR (Smp_153210), MIP. Single FISHes shown in Fig. 2d and 2g, respectively. White arrows: double positive cells. **(g-i)** Double FISH of *actin-2* (Smp_307020) and other muscle cell markers in adult worms. White arrows: double positive cells; green arrowheads: single positive cells expressing genes indicated in green.

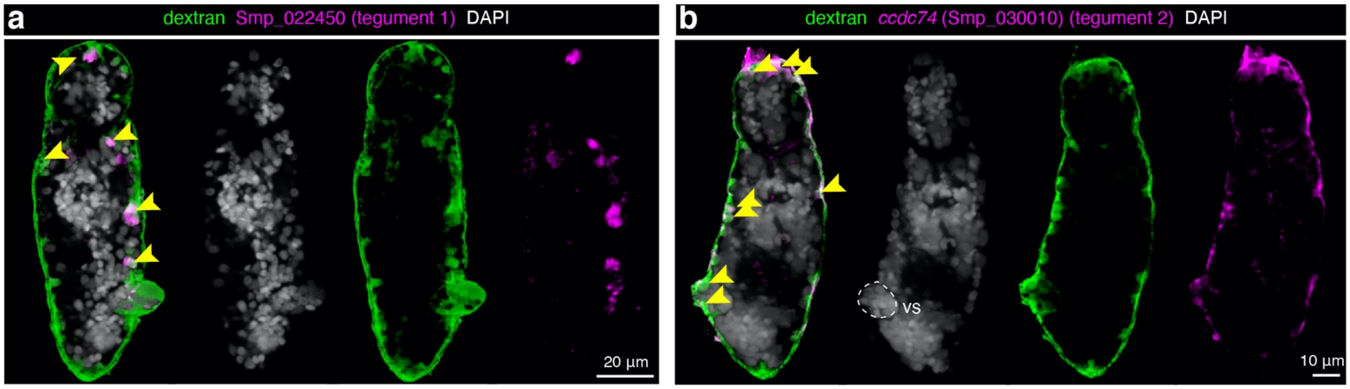

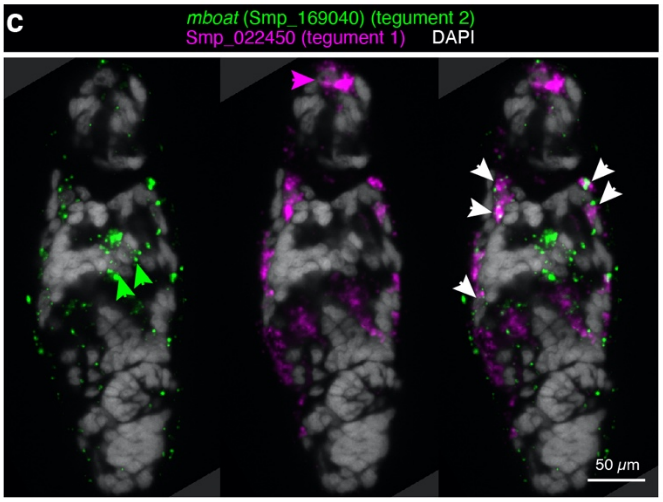

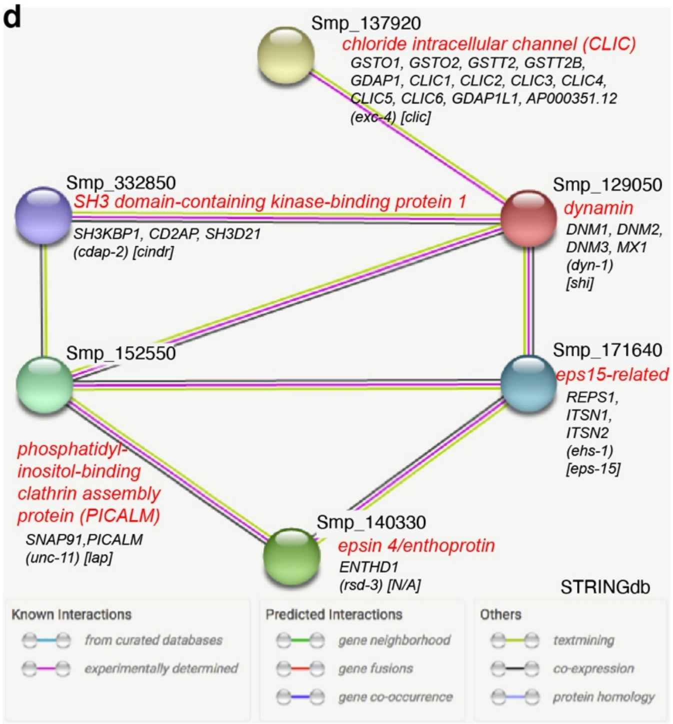

e

Genes	myAUC from Seurat	power	Percentage expression Tegument 2	Percentage expression in the rest of the clusters
Smp_332850	0.703	0.406	0.444	0.044
Smp_171640	0.72	0.44	0.505	0.065
Smp_152550	0.682	0.364	0.515	0.146
Smp_140330	0.723	0.446	0.495	0.064
Smp_129050	0.693	0.386	0.434	0.051
Smp_137920	0.748	0.496	0.636	0.19

Human orthologs from WormBase ParaSite; (*C.elegans* orthologs from WormBase ParaSite); [*D.melanogaster* orthologs from WormBase ParaSite]

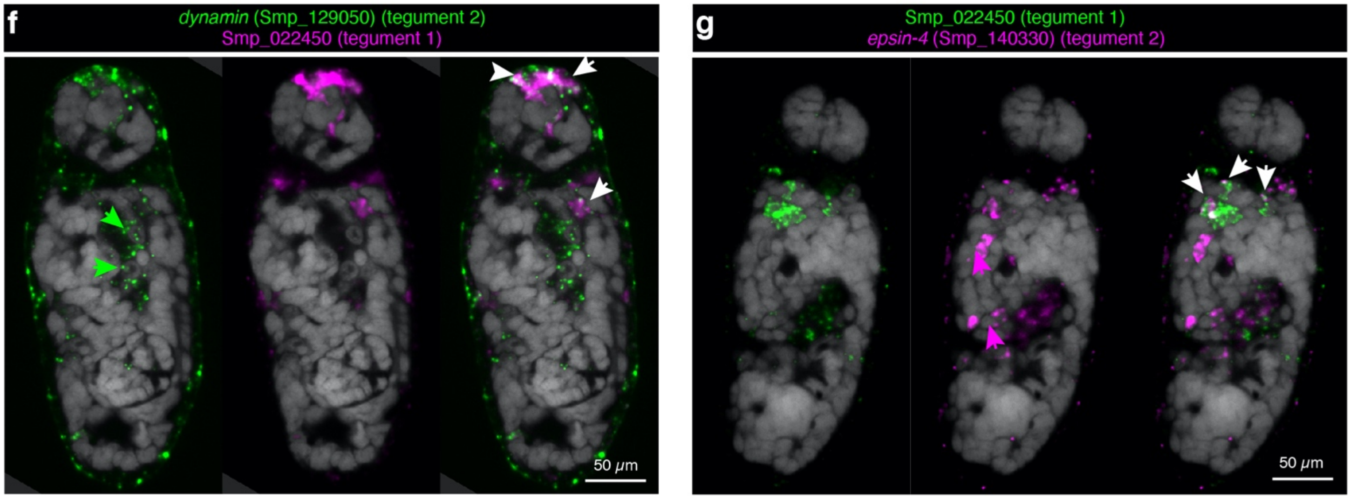

Supplementary Figure 5. (a-b) Dextran labelling in schistosomula shows co-localisation with Tegument 1 and 2 markers. Yellow arrowheads indicate cells positive for both dextran and tegument marker. vs:

ventral sucker. **(c)** Double FISH of Tegument 2 marker *mboat* (Smp_169040) with Tegument 1 marker Smp_022450, single confocal section. White arrow: double positive cell; green and magenta arrows: singly positive cell. **(d-e)** Prediction of biological processes enriched in Tegument 2 relative to Tegument 1. We identified genes that are strong markers (with AUC ≥0.65 in Seurat) for Tegument 2 but not for Tegument 1 (shown in Fig. 3a). The set of genes shown is the largest connected component, i.e. set of predicted interacting genes in the STRINGdb results. The interaction cluster included several genes related to clathrin-mediated (receptor-mediated) endocytosis. These included *phosphatidylinositol-binding clathrin assembly protein (PICALM)*, *Eps15-related*, and epsin-related genes. **(f-g)** Double FISH and single confocal sections of Tegument 2 genes **(f)** *dynamin* and **(g)** *epsin-4*. White arrowhead: double positive cell; green and magenta arrowhead: singly positive cell.

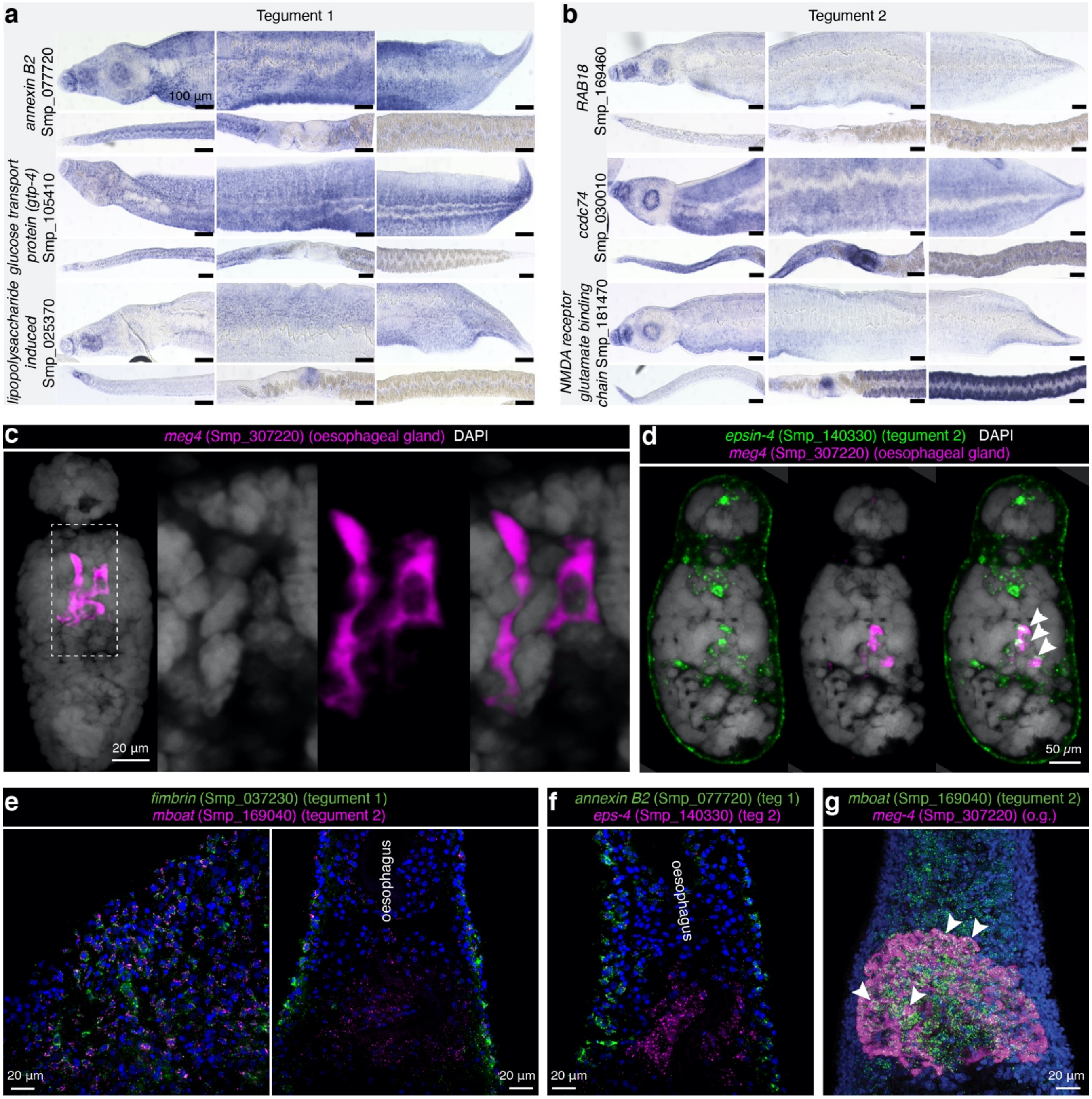

Supplementary Figure 6. (a-b) WISH of Tegument 1 and Tegument 2 maker genes in adult parasites. Scale bar: 100 µm. **(c)** FISH of oesophageal gland marker *meg4* (Smp_307220), MIP. Zoomed-in single confocal section in the dotted box is shown on the right. **(d)** Double FISH of *epsin-4* and *meg-4*, single confocal sections. White arrowhead: double positive cell. **(e-g)** Double FISH of genes indicated in each panel in adult worms. **(e)** *fimbrin* (Smp_037230) and *mboat* (Smp_169040) are co-expressed in the majority of cells, while *mboat* shows enrichment in the oesophagus region, single confocal section. **(f)** *eps-4* (Smp_140330) also shows enrichment in the oesophagus, while co-expressed in *annexin B2*+ cells at a lower level, single confocal section. **(g)** *mboat* (Smp_169040) and *meg-4* (Smp_307220) show co-localisation (few examples highlighted in white arrowheads), MIP.

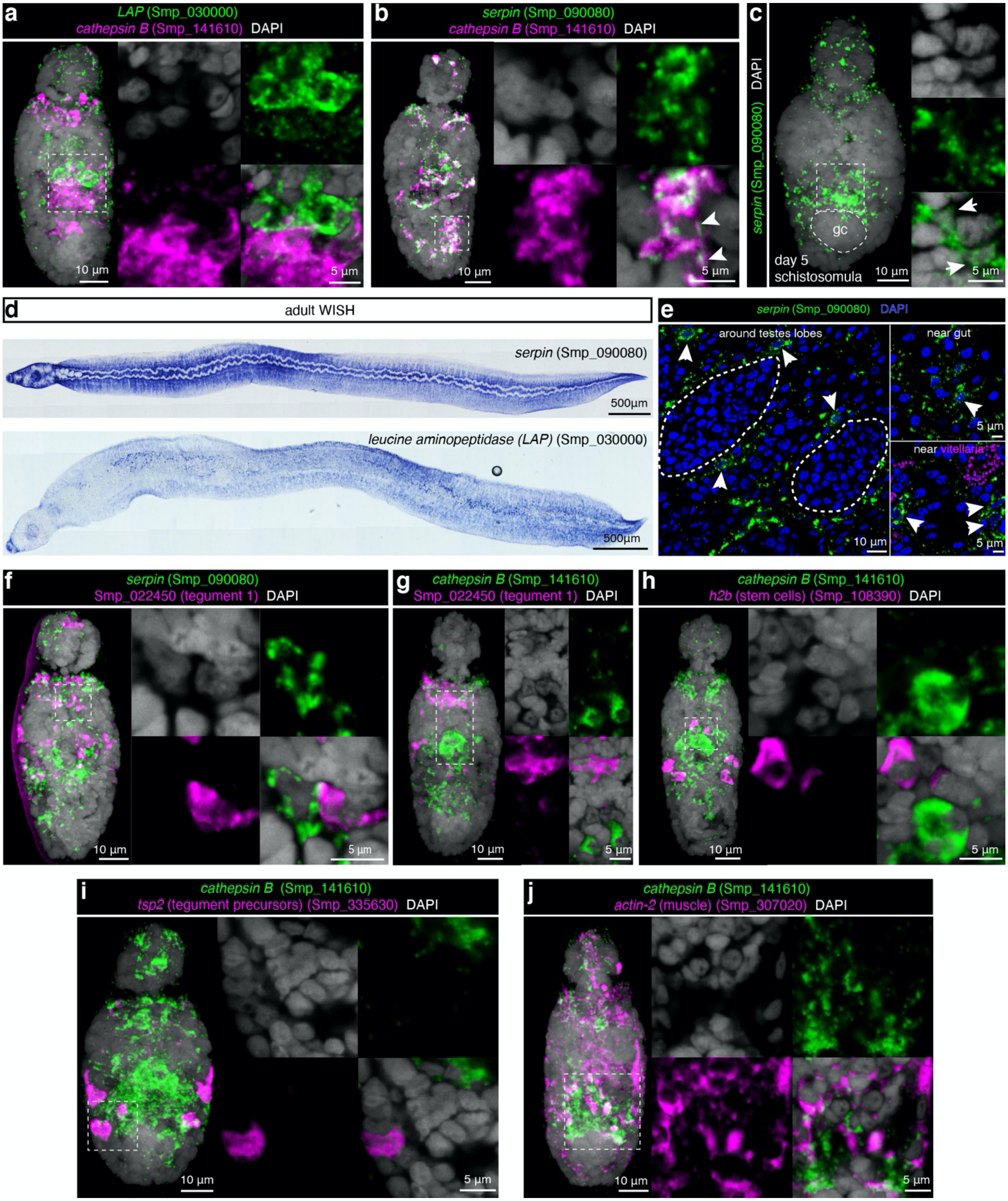

Supplementary Figure 7. (a-b) Double FISH of parenchymal cell marker *lap* (Smp_030000) **(a)** and *serpin* (Smp_090080) **(b)** with *cathepsin B* (Smp_141610). **(c)** *serpin* FISH in five-day old schistosomula. **(a-c)** MIP of whole worm is shown on the left, and a single magnified confocal section from the dotted

box is shown on the right. White arrows indicate a positive cell that has long cytoplasmic processes. **(d)** WISH of *serpin* and *lap* in adult parasites; *lap* is expressed in the worm parenchyma as well as in the gut. **(e)** Single confocal sections showing FISH of *serpin* in different regions of the adult worm. White arrows indicate single positive cells. **(f-j)** Double FISH of parenchymal cell markers and other indicated cell type markers in two-day old schistosomula. Parenchymal cell markers do not co-localise with **(f-g)** tegument cells, **(h)** stem cells, or **(i)** tegument precursors but show some co-localisation with **(j)** muscle cells. MIP is shown for the whole worm on the left, and single confocal sections from the dotted box are shown on the right.

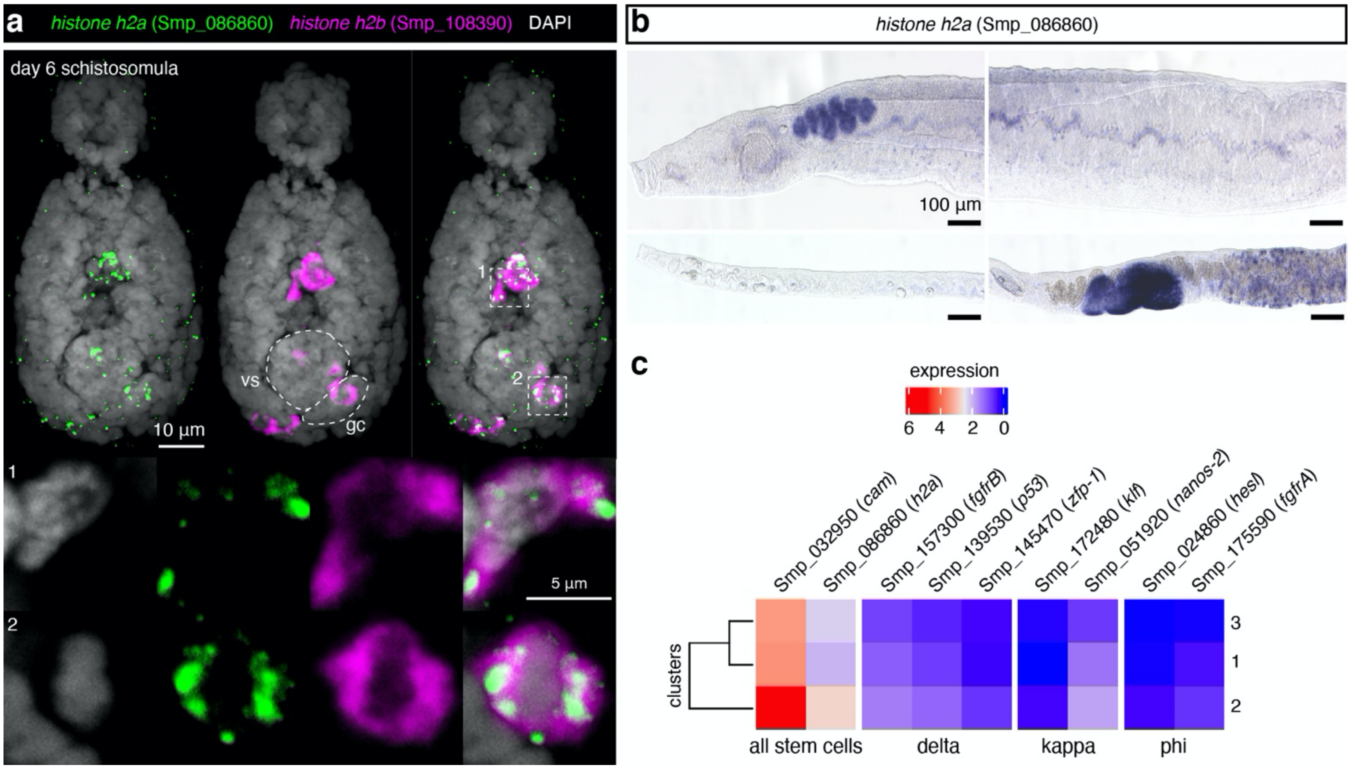

Supplementary Figure 8. (a) Double FISH of *h2a* identified in our dataset, and *h2b*, a known validated schistosome stem cell marker in six-day old schistosomula. *h2a*+ cells co-express *h2b* in both the soma as well as in the germinal cell cluster. Top: MIP for whole worm; Bottom: single confocal magnified sections from the dotted box regions. vs: ventral sucker; gc: germinal cells. **(b)** WISH of *h2a* in adult parasites show expression in gonads and somatic cells, consistent with *calmodulin* and other previously characterised stem cell genes. **(c)** Heatmap of markers identified by Wang, *et al*., 2018. The stem cells were extracted from the dataset and clustered. The markers from Wang, *et al*., 2018 were used to identify three stem cell populations: delta, kappa and phi. No evidence was found to justify the further sub-clustering of these stem cells.

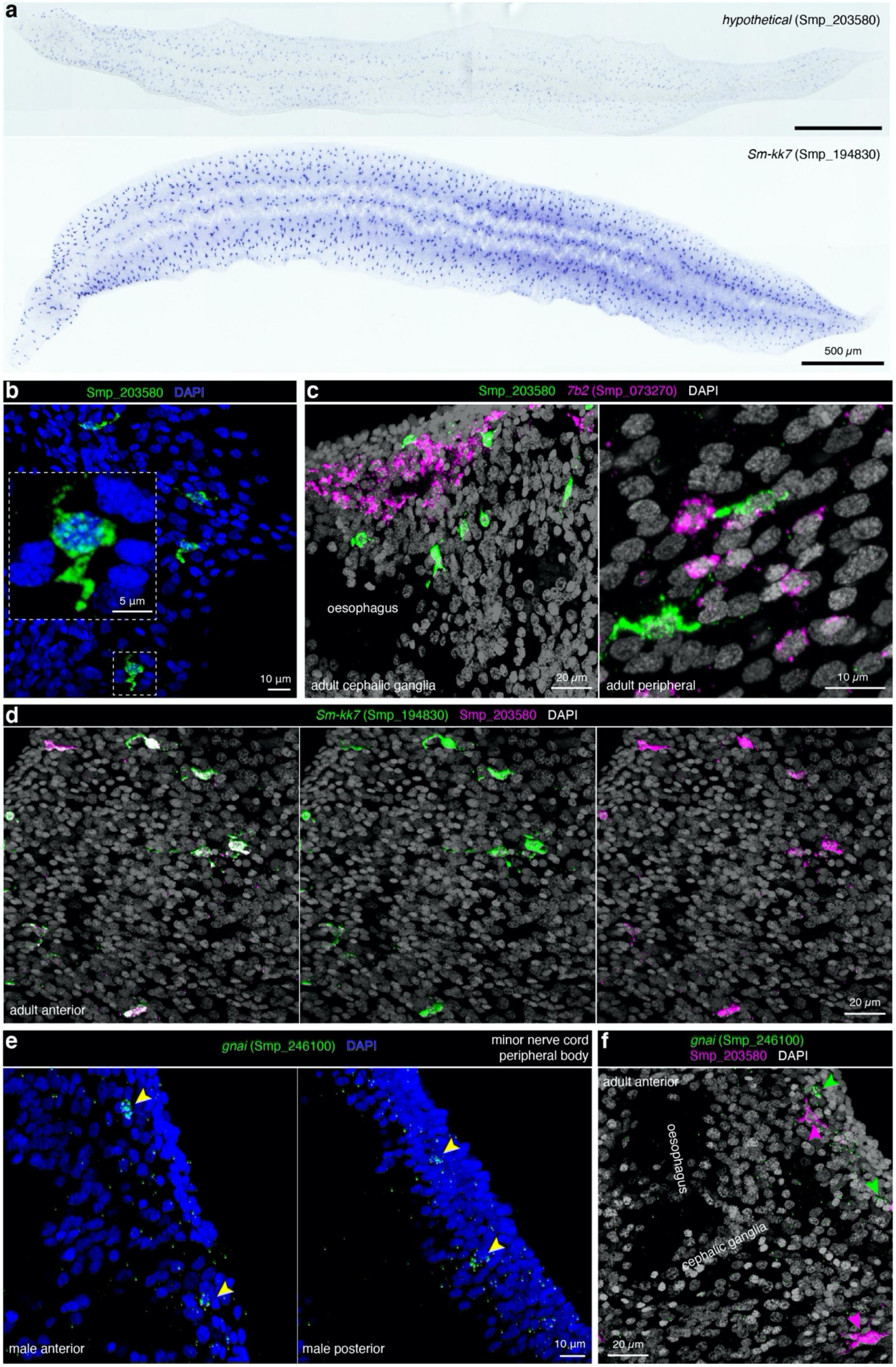

Supplementary Figure 9. (a) WISH of indicated neuronal markers in adult parasites. **(b)** FISH of Smp_203580 in adult male soma (mid-body) shows long cellular processes in each cell. **(c-d)** Double

FISH in adults reveals that Smp_203580 does not co-localise with pan-neuronal marker *7b2* **(c)**, but nearly all cells that express Smp_203580 co-express *Sm-kk7* (white signal) **(d)**. **(e-f)** **(e)** *gnai* is expressed throughout the body of the adult worm but **(f)** does not co-localise with Smp_203580. Green and magenta arrowheads indicate single positive cells for respectively labelled genes.

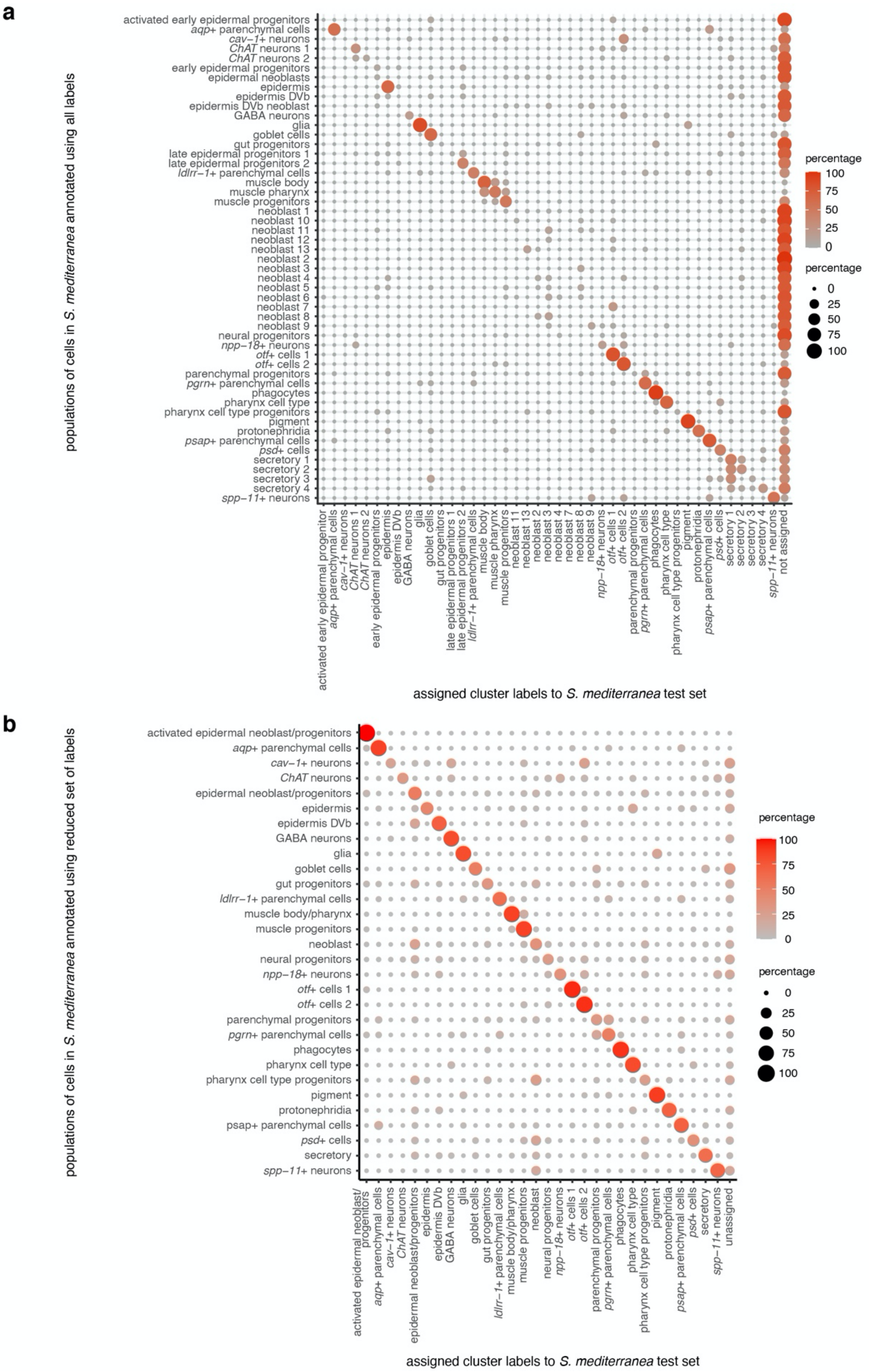

Supplementary Figure 10. Performance of random forest (RF) classifier against *Schmidtea mediterranea* test data. The RF was trained using approximately 70% of the cells from the entire *S.*

mediterranea dataset, with proportional representation from each cluster. The trained RF model was then used to classify each cell in the remaining 30% of the data into learned cluster labels (described in Methods). Each dot represents the percentage of cells from known clusters in the test set that could be successfully categorised using the trained RF model. It is important to note that the assignment is completely agnostic to the labels in the test set. For the test set, each cell was assigned a cluster label if >16% of trees converged onto a majority vote during the prediction analysis. **(a)** Performance using all the cluster labels shown in Plass *et. al.*, 2018. When all labels are used, some cluster labels were never assigned because no cells were confidently predicted to belong to their group. Only 43 cluster labels (out of 51) that had at least one cell assigned to their group with the specified threshold (>16% of trees converging onto a decision for assign a label to that cell) are shown on the x-axis. For some groups shown in the x-axis where cell identities were predicted, the prediction matches the ‘correct’ identity (24 cell labels out of 43). The rest of the groups, the cell identities did not match the expected cell identity. Cells that could not be assigned a cluster label are represented in the ‘not assigned’ category. Based on this figure, we decided to pool groups with similar labels i.e. neoblasts as specified in the Methods **(b)** Performance using a reduced set of labels, including some pooled terms (e.g. neoblast) where the original categories proved to be ambiguous.

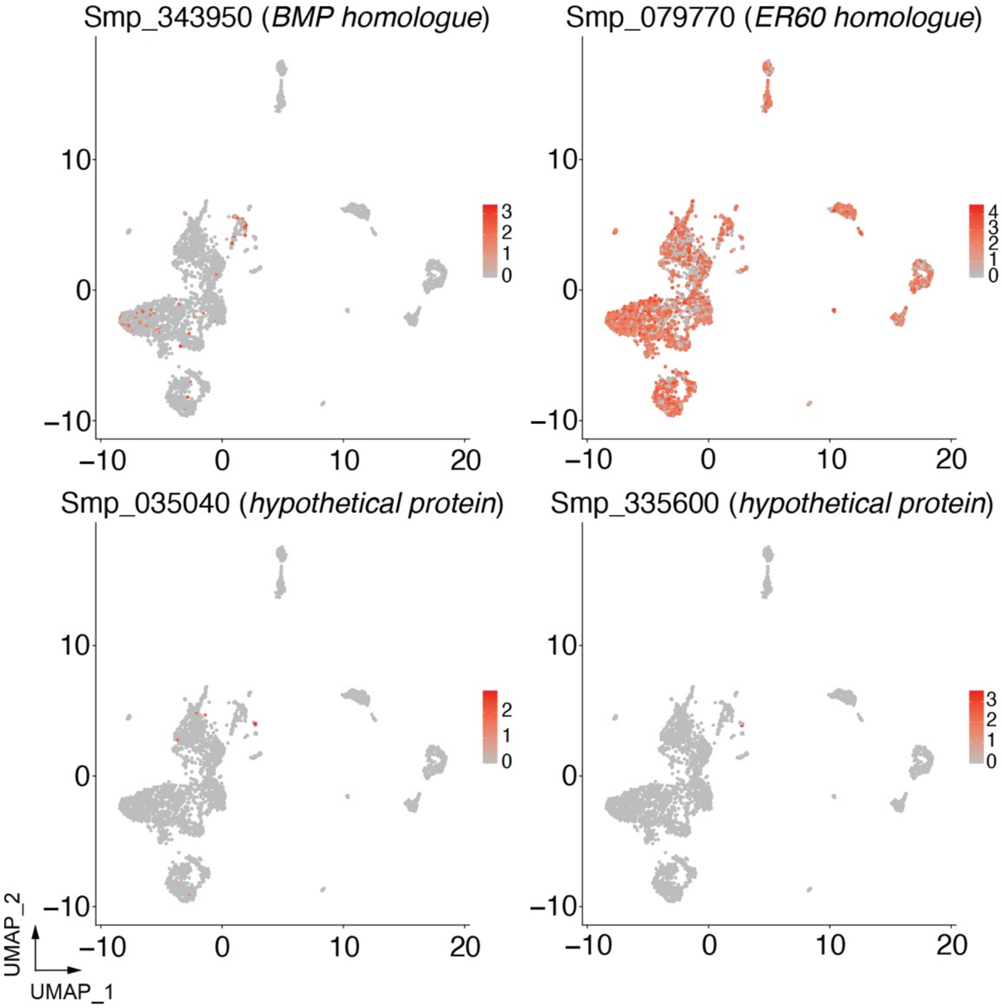

Supplementary Figure 11. UMAP plots showing the expression of protonephridial genes. We show expression for two previously identified protonephridial markers: bone morphogenic protein (BMP) homologue (Smp_343950) and an ER 60 homologue (Smp_079770) and two flame cell markers, hypothetical (Smp_335600) and hypothetical (Smp_035040).

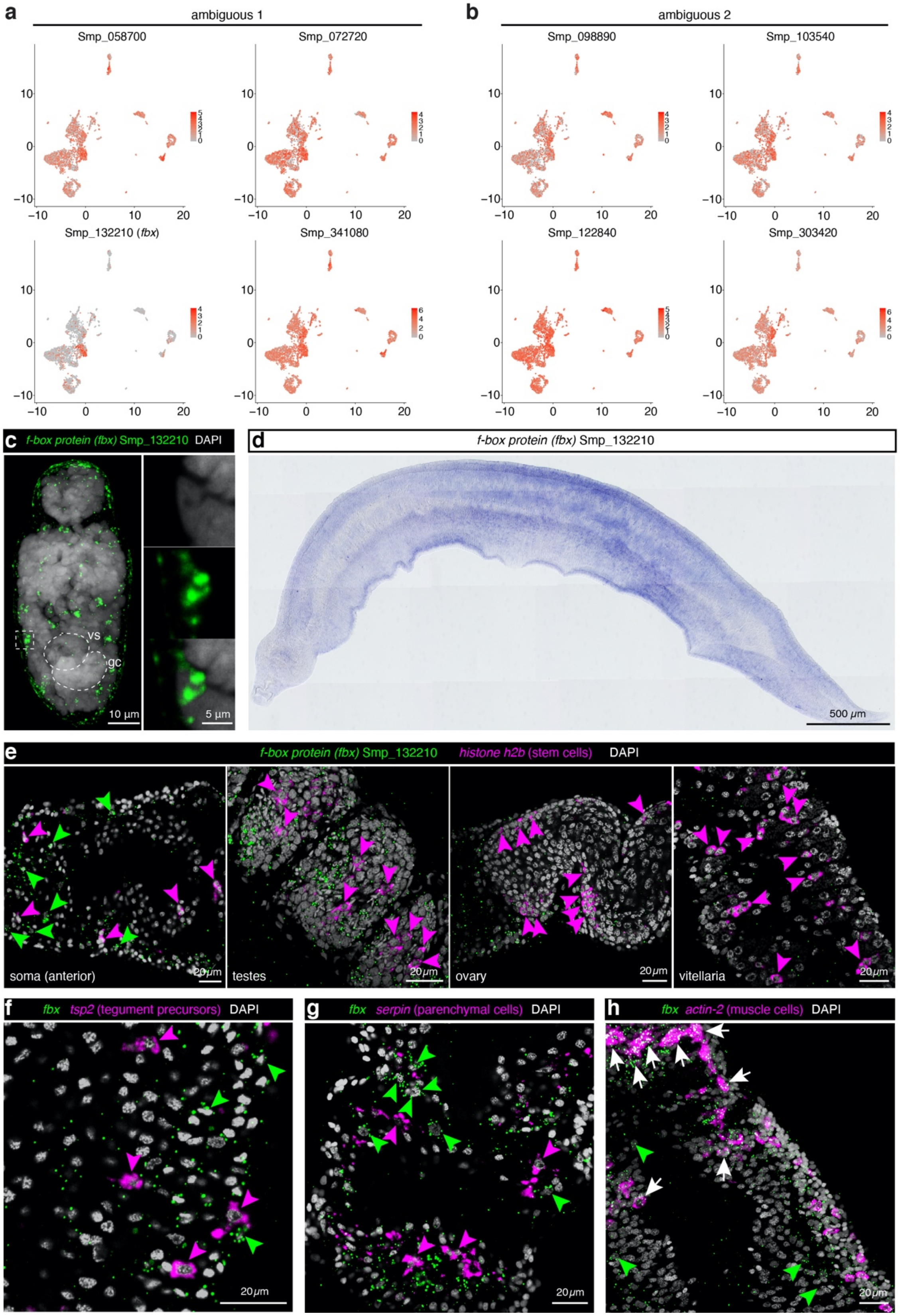

Supplementary Figure 12. Ambiguity of the unidentified clusters. **(a-b)** UMAP plots showing the expression of top marker genes identified by Seurat for the ambiguous clusters highlighting the lack of

specificity, except for only one gene *fbx*; Smp_132210. **(c-d)** ISH validation for the only gene that appears to be specific to ambiguous 1 (*fbx*; Smp_132210) in **(c)** schistosomula and in **(d)** adults. **(e)** Double FISH for *fbx* (Smp_132210) with *histone 2b* marker for stem cells in different parts of the adult tissue. From left to right: soma anterior, testes, ovary and vitellaria. **(f)** Double FISH for *fbx* (Smp_132210) with *tsp2* tegumental marker in the soma of the parasite. **(g)** Double FISH for *fbx* (Smp_132210) with *serpin*, a parenchymal marker. **(h)** Double FISH for *fbx* (Smp_132210) with *actin-2* muscle marker.

Supplementary References

1. Wang, B. *et al.* Stem cell heterogeneity drives the parasitic life cycle of Schistosoma mansoni. *eLife* vol. 7 (2018).
2. Plass, M. *et al.* Cell type atlas and lineage tree of a whole complex animal by single-cell transcriptomics. *Science* **360**, (2018).
