# Supplementary material for: Single-cell atlas of the first intra-mammalian developmental stage of the human parasite Schistosoma mansoni
Source: Nat Commun. 2020 Dec 18;11:6411. doi: 10.1038/s41467-020-20092-5 (PMC7749135; doi:10.1038/s41467-020-20092-5)
Supplement: Supplementary file 3 — Description of Additional Supplementary Files [file 41467_2020_20092_MOESM3_ESM.pdf]

Description of Additional Supplementary Files

File Name: Supplementary Data 1
Description: Sample manifest for the samples used in the study

File Name: Supplementary Data 2
Description: Seurat markers identified by "ROC" test

File Name: Supplementary Data 3
Description: Literature markers used to help identify schistosomula populations

File Name: Supplementary Data 4
Description: Cloning information for all markers used for validation

File Name: Supplementary Data 5
Description: Orthologous genes for S. mediterranea and S. mansoni used in the Random Forest Analysis

File Name: Supplementary Data 6
Description: Manually curated marker genes for representative populations between S. mediterranea (Plass, et al., 2018) and S. mansoni

File Name: Supplementary Data 7
Description: The number of independent in situ hybridization experiments performed
