# Supplementary material for: Single-cell atlas of the first intra-mammalian developmental stage of the human parasite Schistosoma mansoni
Source: Nat Commun. 2020 Dec 18;11:6411. doi: 10.1038/s41467-020-20092-5 (PMC7749135; doi:10.1038/s41467-020-20092-5)
Supplement: Supplementary file 5 — Reporting Summary [file 41467_2020_20092_MOESM5_ESM.pdf]

nature research

Corresponding author(s): Dr. Matthew Berriman
Dr. Gabriel Rinaldi
Prof. Phillip A. Newmark

Last updated by author(s): Oct 14, 2020

Reporting Summary

Nature Research wishes to improve the reproducibility of the work that we publish. This form provides structure for consistency and transparency in reporting. For further information on Nature Research policies, see our Editorial Policies and the Editorial Policy Checklist.

Statistics

For all statistical analyses, confirm that the following items are present in the figure legend, table legend, main text, or Methods section.

n/a	Confirmed	
[ ]	[x]	The exact sample size (*n*) for each experimental group/condition, given as a discrete number and unit of measurement
[ ]	[x]	A statement on whether measurements were taken from distinct samples or whether the same sample was measured repeatedly
[ ]	[x]	The statistical test(s) used AND whether they are one- or two-sided<br>*Only common tests should be described solely by name; describe more complex techniques in the Methods section.*
[x]	[ ]	A description of all covariates tested
[x]	[ ]	A description of any assumptions or corrections, such as tests of normality and adjustment for multiple comparisons
[x]	[ ]	A full description of the statistical parameters including central tendency (e.g. means) or other basic estimates (e.g. regression coefficient) AND variation (e.g. standard deviation) or associated estimates of uncertainty (e.g. confidence intervals)
[ ]	[x]	For null hypothesis testing, the test statistic (e.g. *F*, *t*, *r*) with confidence intervals, effect sizes, degrees of freedom and *P* value noted<br>*Give P values as exact values whenever suitable.*
[x]	[ ]	For Bayesian analysis, information on the choice of priors and Markov chain Monte Carlo settings
[x]	[ ]	For hierarchical and complex designs, identification of the appropriate level for tests and full reporting of outcomes
[x]	[ ]	Estimates of effect sizes (e.g. Cohen's *d*, Pearson's *r*), indicating how they were calculated

Our web collection on statistics for biologists contains articles on many of the points above.

Software and code

Policy information about availability of computer code

Data collection

Schistosoma mansoni
- Dissociated single cells were sorted and analyzed using Flowjo (version vl0.5.3).
- Single-cell libraries were sequenced on an Illumina Hiseq4000 (paired-end reads 75bp).
- Single-cell RNA-seq data were mapped to the S. mansoni reference (v7) using the lOX Genomics analysis pipeline Cell Ranger (v 2.1.0).
- Confocal images were captured using ZEN (ZEISS, LSM880) or Metamorph v7.8.11.0 (Andor, WDb Spinning Disk Confocal).
- Colorimetric in situ hybridization images were captured using ZEN (ZEISS, AxioZoom.V16).

Schmidtea mediterranea
-We did not collect this dataset. Single-cell data comprising 21,610 cells was downloaded from https://shiny.mdc-berlin.de/psca/

Data analysis

Schistosoma mansoni data were further processed as follows:
- Clustering with the Seurat R package (version 3.1.5), using the same matrix of counts
- GO term enrichment using the weightOl and Fisher's Exact Test provided in topGO R package (version v2.34.0). A custom script was used to run the analysis. Script is provided with this document
- STRINGdb vll.O used to identify putative interactions
- OrthoMCL (version vl.4) was used to find orthologues between S. mansoni reference (v7) and schmidtea_mediterranea v6 (from Planmine).
- A custom Python script was used to infer as many 1:1 orthologue clusters as possible and rewrite single cell matrix with dd_Smed identifiers where possible or Smp identifiers as 2nd best option. Script is provided with this document
- Random Forest analysis performed with an in-house script. The script is provided with this document
Schmidtea mediterranea dataset
- Analysed using Seurat package (version 3.1.5)
- Captured images were processed for linear brightness/contrast using Imaris 9.4 (Bitplane).

- Adobe Illustrator/Phostoshop (2020) were used to polish and arrange data/images for manuscript preparation.

For manuscripts utilizing custom algorithms or software that are central to the research but not yet described in published literature, software must be made available to editors and reviewers. We strongly encourage code deposition in a community repository (e.g. GitHub). See the Nature Research guidelines for submitting code & software for further information.

Data

Policy information about availability of data

All manuscripts must include a data availability statement. This statement should provide the following information, where applicable:
- Accession codes, unique identifiers, or web links for publicly available datasets
- A list of figures that have associated raw data
- A description of any restrictions on data availability

The raw data used in this study has been deposited in ENA with accession number PRJEB34071. The individual sample IDs in ENA are the following: ERS3714216 (FUGI_R_D7119553), ERS3714223 (FUGI_R_D7159524) and ERS3714217 (FUGI_R_D7159525). The data has also been deposited in ArrayExpress with accession number E-MTAB-9684. The data can be visualised and navigated from the following website: https://www.schistosomulacellatlas.org/
The code used to analyse the data can be found using the following address: https://zenodo.org/badge/latestdoi/2710309101

Field-specific reporting

Please select the one below that is the best fit for your research. If you are not sure, read the appropriate sections before making your selection.

[x] Life sciences [ ] Behavioural & social sciences [ ] Ecological, evolutionary & environmental sciences

For a reference copy of the document with all sections, see nature.com/documents/nr-reporting-summary-flat.pdf

Life sciences study design

All studies must disclose on these points even when the disclosure is negative.

Sample size	- We analysed 3,513 total cells from the dissociation of the larval S. manosoni schistosomula.<br>- For schistosomula in situ hybridization, 5-10 worms were analysed for each independent experiment.<br>- For adult in situ hybridization, ~5 males and ~5 females were analysed for each independent experiment.
Data exclusions	Low quality cells were filtered out computationally, see Methods
Replication	- Whole schistosomula parasites were collected from two different snail batches and were considered biological replicates (Sample FUGI_R_D7119553 and FUGI_R_D7159524). Data collected as batch 3 (FUGI_R_D7159525) are 'technical' replicates of FUGI_R_D7159524 given they were collected on the same day and from the same pool of parasites (see manifest in Supplementary table 1).<br>- For in situ hybridization in schistosomula, single FISH (fluorescent in situ hybridization) was first performed for each gene and consistency in expression was determined across 5-10 worms. Following single FISH, multiple double FISH experiments were performed with various marker combinations (see Methods for details).<br>- For in situ hybridization in adults, in most cases, we first performed WISH (colorimetric whole-worm in situ hybridization) with ~5 males and ~5 females and confirmed the consistency in expression across animals. Following WISH, we performed multiple single and/or double FISH experiments to confirm the consistency in expression pattern across FISH experiments (see Methods for details).
Randomization	There were no experimental groups, just replicates, therefore randomization is not relevant.
Blinding	We performed an unbiased analysis to all datasets from the different lOX reactions.

Reporting for specific materials, systems and methods

We require information from authors about some types of materials, experimental systems and methods used in many studies. Here, indicate whether each material, system or method listed is relevant to your study. If you are not sure if a list item applies to your research, read the appropriate section before selecting a response.

Materials & experimental systems

n/a	Involved in the study	
[ ]	[x]	Antibodies
[x]	[ ]	Eukaryotic cell lines
[x]	[ ]	Palaeontology and archaeology
[ ]	[x]	Animals and other organisms
[x]	[ ]	Human research participants
[x]	[ ]	Clinical data
[x]	[ ]	Dual use research of concern

Methods

n/a	Involved in the study	
[x]	[ ]	ChIP-seq
[ ]	[x]	Flow cytometry
[x]	[ ]	MRI-based neuroimaging

Antibodies

Antibodies used	anti-DIG-POD (MilliporeSigma, 11207733910); anti-FITC-POD (MilliporeSigma, 11426346910); anti-DNP-HRP (Vector Laboratories, custom); anti-DIG-AP (MilliporeSigma, 11093274910).
Validation	- anti-DIG-POD (https://www.sigmaaldrich.com/catalog/product/roche/11207733910?lang=en&region=US)<br>General description: Digoxigenin is a hapten, useful in labeling nucleic acids and in detection systems. This product contains Fab fragments from an anti-digoxigenin antibody, conjugated with horse-radish peroxidase (POD). Anti-Digoxigenin-POD, Fab fragments is useful for the detection of digoxigenin-labeled compounds.<br>Specificity: The polyclonal antibody from sheep is specific to digoxigenin and digoxin and shows no cross-reactivity with other steroids, such as human estrogens and androgens.<br><br>- anti-FITC-POD (https://www.sigmaaldrich.com/catalog/product/roche/11426346910?lang=en&region=US)<br>General Description: Fab fragments from polyclonal anti-fluorescein antibodies, conjugated to horseradish peroxidase. The reagent is an anti-fluorescein antibody, Fab fragments from sheep, conjugated with horse-radishperoxidase (POD). After immunization with fluorescein the sheep IgG (immunoglobulin G) was purified by ion exchange chromatography and the specific IgG was isolated by immunosorption. The Fab fragments obtained by papain digestion were purified by gel filtration, conjugated with POD and stabilized in 60mM Tris-Hepes-buffer, 0.4% bovine immunoglobulin (w/v), 0.2% Germall II (w/v), pH 7.2.<br>- Specificity: The polyclonal antibody reacts with free and bound fluorescein.<br><br>- anti-DNP-HRP (https://vectorlabs.com/anti-dnp-dinitrophenyl-unconjugated.html)<br>This antibody is not listed on the company's product list. The unconjugated anti-DNP which has been validated and is available for purchase (https://vectorlabs.com/anti-dnp-dinitrophenyl-unconjugated.html) has been tagged with HRP by the company per request from the Newmark Lab. To validate the antibody, we first tested side-by-side anti-DNP (the same unconjugated antibody) conjugated with AP (Alkaline Phosphatase) (MB-3100, Vector Laboratories) and anti-DNP-HRP against a same gene and compared the expression pattern between the colorimetric (Alkaline Phosphatase) and fluorescence (HRP) reactions. We then further validated by generating a riboprobe against a gene in which its expression is known using different labeled UTP nucleotides (DIG, DNP, and FITC). We performed in situ hybridization using antibodies specific to different nucleotide labels (anti-DIG-POD, anti-DNP-HRP, and anti-FITC-POD), and cross checked the expression pattern between the three.<br><br>- anti-DIG-AP (https://www.sigmaaldrich.com/catalog/product/roche/11093274910?lang=en&region=US)<br>General description: Digoxigenin is a hapten, useful in labeling and detection of nucleic acids.[8] This product contains Fab fragments from polyclonal anti-digoxigenin antibodies, conjugated to alkaline phosphatase. Anti-Digoxigenin-AP, Fab fragments are useful for the detection of digoxigenin-labeled compounds.<br>Specificity: The polyclonal antibody from sheep is specific to digoxigenin and digoxin and shows no cross-reactivity with other steroids, such as human estrogens and androgens.

Animals and other organisms

Policy information about studies involving animals; ARRIVE guidelines recommended for reporting animal research

Laboratory animals	- The complete life cycle of Schistosoma mansoni (NMRI strain) is maintained at the Wellcome Sanger Institute (WSI).<br>- To maintain the cycle, we used mice (Balb/C female mice, 8 to 12 weeks old by the time of infection) as definitive hosts. The mouse infections at WSI were conducted under Home Office Project Licence No. P77E8A062 held by Dr. Gabriel Rinaldi, and all protocols were presented and approved by the Animal Welfare and Ethical Review Body (AWERB) of the WSI, and Institutional Animal Care and Use Committees (IACUC) at the University of Wisconsin-Madison (protocol M005569). The AWERB is constituted as required by the UK Animals (Scientific Procedures) Act 1986 Amendment Regulations 2012. To harvest parasites for validation using in situ hybridization, we used Swiss-Webster (Taconic Biosciences) female mice that are between 5 to 12 weeks of age at the time of infection. The mouse infection was done using S. mansoni (NMRI strain received from Biomedical Research Institute (Rockville, MD)) and all mice were handled in accordance with the Institutional Animal Care Use Committee protocol at the University of Wisconsin–Madison (M005569)
Wild animals	Study did not involve wild animals
Field-collected samples	Study did not involve samples collected in the field
Ethics oversight	Home Office Project Licence No. P77E8A062<br>Animal Welfare and Ethical Review Body (AWERB) of the WSI<br>Institutional Animal Care and Use Committees (IACUC) at the University of Wisconsin-Madison (protocol M005569). The AWERB is constituted as required by the UK Animals (Scientific Procedures) Act 1986 Amendment Regulations 2012.

Note that full information on the approval of the study protocol must also be provided in the manuscript.

Flow Cytometry

Plots

Confirm that:

- [x] The axis labels state the marker and fluorochrome used (e.g. CD4-FITC).
- [x] The axis scales are clearly visible. Include numbers along axes only for bottom left plot of group (a 'group' is an analysis of identical markers).
- [x] All plots are contour plots with outliers or pseudocolor plots.
- [x] A numerical value for number of cells or percentage (with statistics) is provided.

Methodology

Sample preparation	- Schistosoma mansoni schistosomula were dissociated into single cells<br>- The resulting suspension was passed through 70μm and 40μm cells strainers<br>- Dissociated cells were spun at 300 rpm for 5 mins and resuspended in 1x cold PBS supplemented with 20% heat inactivated fetal bovine serum (twice).<br>- The resulting cell suspension was co-stained with 0.5μg/ml of Fluorescein Diacetate (FDA; Sigma F7378) to label live cells, and lμg/ml of Propidium Iodide (PI; Sigma P4864) to label dead/dying cells<br>- Live cells were sorted into eppendorf tubes by enriching for FDA+/ PI- cells
Instrument	8D Influx™ cell sorter
Software	Flowjo (version vl0.5.3)
Cell population abundance	We have that cells dissociated with Liberase DL had 81.8% of FDA+/PI- cells live cells<br>Cells dissociated with Pronase had 49.6% FDA+/PI- cells live cells
Gating strategy	For gating the cells we first use the FSC/SSC to locate the cells. To determine live/dead cells, we included a negative control where no dyes (PI or FDA was added to the dissociated cell pool). We then used our sample and gated on live cell which were Pland<br>FDA+

- [x] Tick this box to confirm that a figure exemplifying the gating strategy is provided in the Supplementary Information.
